# Clinical Characteristics and Outcomes of Endophthalmitis Before and During the COVID-19 Pandemic

**DOI:** 10.18502/jovr.v18i3.13777

**Published:** 2023-07-28

**Authors:** Blake H Fortes, Prashant D Tailor, Timothy T Xu, Robert A Churchill, Matthew R Starr

**Affiliations:** ^1^Department of Ophthalmology, Mayo Clinic College of Medicine, Rochester, MN, USA; ^2^Alix School of Medicine, Mayo Clinic, Rochester, MN, USA

**Keywords:** COVID-19, Endophthalmitis, Intravitreal injections

## Abstract

**Purpose:**

To evaluate the clinical characteristics and visual acuity outcomes of patients who presented with endophthalmitis prior to and during the coronavirus disease 2019 (COVID-19) pandemic.

**Methods:**

This multicenter retrospective case series with historical controls included consecutive patients presenting with any form of endophthalmitis from March 1, 2019 to September 1, 2019 (pre-COVID-19) and from March 1, 2020 to September 1, 2020 (COVID-19) at Mayo Clinic Rochester (MCR), Health System (MCHS), Arizona (MCA), and Florida (MCF) sites. Cases were divided into “pre-COVID-19” versus “COVID-19” groups depending on when they first presented with endophthalmitis.

**Results:**

Twenty-eight cases of endophthalmitis presented to all Mayo Clinic sites during the study period. Of these, 10 patients presented during the first six months of the COVID-19 pandemic. During the same six-month period the year prior, 18 patients presented with endophthalmitis. Endophthalmitis etiology (post-injection, post-cataract extraction, post-glaucoma filtering surgery, post-pars plana vitrectomy, endogenous, and others) was similar between both groups (*P* = 0.34), as was post-injection endophthalmitis rate (*P* = 0.69), days to presentation (*P* = 0.07), initial management (*P* = 0.11), culture-positivity rate (*P* = 0.70), and need for subsequent pars plana vitrectomy (*P* = 1). Visual acuity outcomes were similar between both groups at six months, however, the mean LogMAR visual acuity at presentation was worse in the COVID-19 group compared to the pre-COVID-19 group (2.44 vs 1.82; *P* = 0.026).

**Conclusion:**

Clinical characteristics and the post-injection endophthalmitis rate were similar during both periods, however, patients presented with worse vision during the pandemic suggesting that the pandemic may have contributed to delayed presentation, regardless, outcomes are still poor.

##  INTRODUCTION

The coronavirus disease 2019 (COVID-19) pandemic had profound impacts on the practice of medicine globally with numerous medical societies making recommendations to postpone all elective visits and surgeries, including the American Academy of Ophthalmology, the Royal College of Ophthalmologists, and the Asia-Pacific Academy of Ophthalmology, among others.^[1,2,3]^ Ophthalmology, which largely involves elective surgeries and outpatient appointments, is one of the medical specialties with the highest risk of COVID-19 infection given the time spent in close proximity to the patient during the examination and possible conjunctival transmission.^[4,5]^ As a result, ophthalmology has experienced significant pandemic-related impact, with > 90% decrease in medical and surgical volume during the beginning of the COVID-19 pandemic.^[6,7,8,9]^ Fear of COVID-19 exposure and other factors also changed patients' ability and willingness to attend their regularly scheduled follow-up appointments leading to high rates of cancellation and missed outpatient appointments.^[10,11,12,13,14,15,16,17,18]^ In certain conditions requiring regular intravitreal injections for preservation of vision or other urgent/emergent conditions, these delays may potentially lead to irreversible loss of vision, as in neovascular age-related macular degeneration (nAMD) resulting in submacular hemorrhage, or more severe pathology necessitating more complex surgical intervention, as in rhegmatogenous retinal detachment (RRD).^[19,20,21,22,23]^

Endophthalmitis is another example of an emergent vision-threatening condition requiring prompt diagnosis and treatment to preserve vision. Only one prior study by Das et al has investigated the impact of the COVID-19 pandemic on endophthalmitis presentation.^[24]^ This was a cross-sectional study comparing the distribution of patients with endophthalmitis in India who presented during the COVID-19 lockdown and unlock periods with patients who presented in the three years preceding the pandemic. This study found that there was a > 50% decline in the number of patients who presented with endophthalmitis. Additionally, there was an increase in the percentage of patients who presented with endogenous endophthalmitis and a decrease in post-traumatic endophthalmitis. However, this study did not investigate clinical characteristics, days to presentation, or visual acuity (VA) outcomes of patients with endophthalmitis.^[24]^

Our study aims to evaluate the clinical characteristics and VA outcomes of patients who presented with endophthalmitis of any etiology prior to and during the initial six-month period of the COVID-19 pandemic.

##  METHODS

This multi-center, retrospective case series received approval from the Mayo Clinic institutional review board. The study conformed to the tenets of the Declaration of Helsinki and data were collected in accordance with the Health Insurance Portability and Accountability Act of 1996 guidelines.

Study subjects who presented with endophthalmitis of any etiology were selected. Patients were included if they presented with any form of endophthalmitis from March 1, 2019 to September 1, 2019 (pre-COVID-19 cohort) and from March 1, 2020 to September 1, 2020 (COVID-19 cohort) at Mayo Clinic Rochester (MCR), Mayo Clinic Arizona (MCA), Mayo Clinic Florida (MCF), or Mayo Clinic Health System (MCHS) sites during either time period. Cases were divided into “pre-COVID-19” cases versus “COVID-19” cases depending on the initial date of endophthalmitis presentation. Cases were excluded if they did not present during either time interval. Patients were identified via endophthalmitis International Classification of Diseases-10 (ICD-10) billing codes. These patients' medical records were individually reviewed to confirm the endophthalmitis diagnosis.

For patients confirmed to have developed endophthalmitis of any etiology during either time interval, we obtained the following variables: demographics, endophthalmitis etiology (post-intravitreal injection, post-cataract extraction, post-glaucoma filtering surgery, post-pars plana vitrectomy (PPV), endogenous, or other causes such as post-traumatic or related to corneal ulceration), days to presentation, initial management (tap and inject, PPV, or medical management), subsequent PPV, signs and symptoms at presentation, Snellen best corrected VA at most recent visit prior to presentation, and VA at presentation. If available, we recorded VA at the following time points: at six months, at one-year, and final VA. We also included the results of vitreous/aqueous gram stain and culture, and follow-up duration. For post-injection endophthalmitis cases, the intravitreal agent and injection indication were reviewed.

### Endophthalmitis Treatment Protocol

The diagnosis of endophthalmitis was based on a characteristic presentation of decreased vision, ocular pain, and intraocular inflammation. Affected patients underwent initial treatment with one of the following: intravitreal antibiotic injection with vitreous/aqueous tap, PPV with vitreous tap and intravitreal injection of antibiotics, or medical treatment with cycloplegic and corticosteroid eye drops at the treating retinal specialist's discretion. Patients were usually treated with intravitreal ceftazidime (2.25 mg/0.1 mL) and vancomycin (1.0 mg/0.1 mL) at endophthalmitis presentation. Patients with a penicillin allergy were treated with amikacin (0.4 mg/0.1 mL) instead of ceftazidime. Topical medication regimen consisting of corticosteroid, cycloplegic, and antibiotic eye drops were variable and based on the preferences of the treating physician. All patients were closely followed.

### Statistical Analysis

Statistical analysis was performed utilizing RStudio: Integrated Development for R (RStudio, Inc., RStudio Team (2018), Boston, Massachusetts, https://www.rstudio.com). The primary outcomes were the fraction of endophthalmitis etiology, days to presentation, initial management, subsequent PPV, and VA outcomes between the pre-COVID-19 and COVID-19 groups. Secondary outcomes included the microbial spectrum, and the rate of post-injection endophthalmitis between the pre-COVID-19 versus the COVID-19 cohorts. Snellen VA was converted to the logarithm of the minimum angle of resolution (LogMAR) VA for statistical analysis. Vision levels of count fingers, hand motion, light perception, and no light perception were assigned LogMAR values of 2.3, 2.6, 2.9, and 3.2, respectively, as established by prior studies.^[25]^ Fisher's exact test was employed to compare categorical variables between the pre-COVID-19 group and the COVID-19 group. A Wilcoxon rank sum test was performed to compare VA outcomes, days to presentation, and follow-up duration. Statistical significance was determined based on an alpha level of < 0.05.

##  RESULTS

Overall, this multicenter retrospective case series identified 28 patients who presented with endophthalmitis of any etiology throughout the study period. Of these, 10 patients presented during the first six months of the COVID-19 pandemic (COVID-19 cohort). During the same six-month period the year prior, 18 patients presented with endophthalmitis (pre-COVID-19 cohort). There were no differences in endophthalmitis etiology (*P *= 0.34), days to presentation (mean days to presentation: COVID-19: 18 days vs pre-COVID-19: 7 days; *P *= 0.07), initial management of endophthalmitis (*P *= 0.11), or subsequent PPV (*P *= 1.0) between the pre-COVID-19 cohort and the COVID-19 cohort [Table 1]. Women were more likely than men to present during the first six months of the COVID-19 pandemic than the same time period the year prior (*P *= 0.016).

Of the cases sent for culture in the pre-COVID-19 group, 6 of 16 (38%) tested culture-positive (3 S*taphylococcus epidermidis*, 1 *Streptococcus agalactiae*, 1 *Cutibacterium acnes*, and 1 *Fusarium falciformis*) compared with 4 of 10 (40%) [1 *S. aureus*, 1 *S. pneumoniae*, 1 *Moraxella nonliquefaciens*, and 1 *Candida albicans*) in the COVID-19 group (*P *= 0.70). In the pre-COVID-19 group, *F.* *falciformis* was isolated in a patient with a corneal ulcer after a penetrating keratoplasty. The case of *C. albicans* in the COVID-19 cohort occurred in a patient who had endogenous endophthalmitis related to a recent history of nephrolithiasis complicated by pyelonephritis.

The mean VA was similar between both groups at baseline prior to endophthalmitis presentation (Pre-COVID-19 LogMAR VA: 0.68 [Snellen VA: 20/96] vs. COVID-19 LogMAR VA: 0.41 [Snellen VA: 20/51]; *P *= 0.59) [Table 2]. Likewise, VA outcomes were similar between the pre-COVID-19 group and the COVID-19 group at six months (Pre-COVID-19 LogMAR VA: 0.92 [Snellen VA: 20/166] vs COVID-19 LogMAR VA: 0.92 [Snellen VA: 20/166]; *P *= 0.97), and at last follow-up (Pre-COVID-19 LogMAR VA: 0.87 [Snellen VA: 20/148] vs COVID-19 LogMAR VA: 1.25 [Snellen VA: 20/355]; *P* = 0.51). However, during the COVID-19 pandemic, patients with endophthalmitis presented with worse VA compared to patients during the pre-COVID-19 era (Pre-COVID-19 LogMAR VA: 1.82 [Snellen VA: 20/1321] vs COVID-19 LogMAR VA: 2.44 [Count fingers]; *P *= 0.026).

Overall, there were 13,761 anti-vascular endothelial growth factor (anti-VEGF) intravitreal injections performed during the pre-COVID-19 era compared to 12,145 during the first six months of the COVID-19 pandemic. There was no difference in the post-injection endophthalmitis rate between these two cohorts (COVID-19: 1 in 3036 injections vs pre-COVID-19: 1 in 1966 injections; *P *= 0.69) [Table 1].

**Table 1:** Clinical characteristics of endophthalmitis cases that presented before the COVID-19 pandemic compared to those during the COVID-19 pandemic.


	**Pre-COVID-19 ** ***n*** ** = 18 (%)**	**COVID-19 ** ***n*** ** = 10 (%)**	***P*** **-values**	**Total ** ***N*** ** = 28 (%)**
Age at presentation (yr)		
Mean (median, range)	72 (70, 53–89)	73 (73, 51–90)	0.72	72 (73, 51–90)
Sex		
Male	11 (61)	1 (10)	12 (43)
Female	7 (39)	9 (90)	0.016	16 (57)
Affected eye		
Right	10 (56)	7 (70)	17 (61)
Left	7 (39)	2 (20)	9 (32)
Both	1 (5)	1 (10)	0.48	2 (7)
Days to Presentation		
Mean (median, range)	7 (5, 1–34)	18 (7, 3–45)	0.07	11 (7, 1–45)
Endophthalmitis Etiology		0.34	
Post-injection	7 (39)	4 (40)	1	11 (39)
Post-cataract extraction	1 (6)	1 (10)	1	2 (7)
Post-glaucoma filtering surgery	1 (6)	2 (20)	0.28	3 (11)
Post-pars plana vitrectomy	0 (0)	1 (10)	0.36	1 (4)
Endogenous	7 (39)	1 (10)	0.19	8 (29)
Others	2 (11)	1 (10)	1	3 (11)
If post-injection, intravitreal injection agent			
Bevacizumab	5 (71)	2 (50)	7 (64)
Ranibizumab	0 (0)	1 (25)	1 (9)
Aflibercept	2 (29)	1 (25)	0.81	3 (27)
Post-injection endophthalmitis	7 (0.0509%)	4 (0.0329%)	11 (0.0425%)
	1 in 1966 injections	1 in 3036 injections	0.69	1 in 2355 injections
Positive vitreous or aqueous culture		
Yes	6 (38)	4 (40)	10 (38)
No	10 (63)	6 (60)	0.7	16 (62)
Initial management		
Intravitreal tap and injection	18 (100)	8 (80%)	26 (93)
Pars plana vitrectomy	0 (0)	1 (10%)	1 (4)
Medical treatment	0 (0)	1 (10%)	0.11	1 (4)
Subsequent pars plana vitrectomy		
Yes	3 (17)	2 (20%)	5 (18)
No	15 (83)	8 (80%)	1	23 (82)
Follow-up duration (months)		
Mean (median, range)	19.8 (26.7, 0.1–32.3)	13.7 (16.1, 1.6–21.5)	0.11	17.5 (18.5, 0.1–32.3)
	
	
*Bold values indicate significant *P*-value.				

**Table 2:** Visual acuity outcomes of endophthalmitis cases that presented before the COVID-19 pandemic compared to those during the COVID-19 pandemic.


**Visual Acuity**	**Pre-COVID-19**	**COVID-19 **	***P*** **-values**
	***n*** ** = 18**	***n*** ** = 10**	
Mean baseline LogMAR VA (Snellen VA)	0.68 (20/96)	0.41 (20/51)	0.59
Mean LogMAR VA at endophthalmitis presentation (Snellen VA)	1.82 (20/1321)	2.44 (CF)	0.026
Mean LogMAR VA at 6 months (Snellen VA)	0.92 (20/166)	0.92 (20/166)	0.97
Mean LogMAR VA at last follow-up (Snellen VA)	0.87 (20/148)	1.25 (20/355)	0.51
	
	
LogMAR, Logarithm of the minimum angle of resolution; VA, visual acuity ${\;}^{*}$ Bold values indicate significant *P*-value.			

##  DISCUSSION

This retrospective multi-center case series evaluated the clinical characteristics and VA outcomes of patients who presented with endophthalmitis of any etiology during the initial six months of the COVID-19 pandemic compared with patients who presented during the same six-month period the year prior. We found that patients who presented with endophthalmitis during the COVID-19 pandemic had worse VA compared to those who presented prior to the pandemic. Although the difference in days to presentation between both groups was not statistically significant (mean days to presentation: COVID-19: 18 days vs pre-COVID-19: 7 days; *P *= 0.07), the sample size was quite limited suggesting that this study may have been underpowered. Taken together, these findings support our hypothesis that the initial phase of the COVID-19 pandemic led to delayed presentation of endophthalmitis with worse VA at presentation compared to cases that presented prior to the pandemic.

Interestingly, delayed presentation during the pandemic did not translate to worse VA outcomes either at six months or at final follow-up, as there were similar outcomes between both groups. This contrasts with other studies investigating the effect of delays in anti-VEGF treatment for patients with neovascular AMD, diabetic macular edema (DME), and central retinal vein occlusion (CRVO), which have demonstrated that delays in care led to worse final VA outcomes.^[19,26,27,28,29]^ In addition, this study did not identify a difference in the post-injection endophthalmitis rate during the pandemic compared to that of the previous year, corroborating a prior IRISⓇ Registry (Intelligent Research in Sight) study's findings.^[30]^ It is possible that no differences in VA were found due to the poor outcomes that generally come from patients who develop endophthalmitis. Even with delayed presentations, there appeared to be no difference in VA.

Endophthalmitis etiology was similar between the pre-COVID-19 and COVID-19 groups both in terms of overall distribution of cases by etiology, but also by comparing rates of each etiology individually. It is important to note, however, that although intravitreal injection volume was similar between both groups, there was a remarkable reduction in surgical volume for elective surgeries, including cataract surgery, and glaucoma surgery, which likely impacted the number of patients who presented with endophthalmitis during the first six months of the COVID-19 pandemic. Similarly, there was only one patient who presented with endogenous endophthalmitis during the COVID-19 pandemic compared to the seven patients in the pre-COVID-19 group. However, this was not a statistically significant difference. There was a similar rate of culture-positive results in both the pre-COVID-19 cohort and the COVID-19 cohort with a similar microbial spectrum in both groups. However, it was interesting to note a case of Moraxella endophthalmitis, an atypical cause for endophthalmitis, in the COVID-19 cohort as well as a case of endogenous Candida infection.^[31]^ It is now known that there appears to be a correlation of candidemia with COVID-19 infection, with reports of endophthalmitis seen in these patients.^[32,33,34]^ However, our patient was not infected with COVID-19 at the time of her infection. Certainly, as the COVID-19 pandemic continues, ophthalmologists should be cognizant of the risk of atypical infections with microbes normally confined to the oral mucosa, but potentially dispersed with poor-fitting or un-taped mask use with air reflux toward the ocular surface as well as fungal endogenous endophthalmitis in patients with superimposed COVID-19 infections.

This study is inherently limited by its retrospective design. Additional limitations of this analysis include the fact that this study did not investigate endophthalmitis clinical characteristics during other periods of the COVID-19 pandemic as policies became less stringent. Inherently, there also is selection bias as patients who presented during the first six months of the COVID-19 pandemic were more likely to have had severe disease compared to those who presented prior to the pandemic. Comparing VA of patients with endophthalmitis is also difficult to interpret as many patients present with different variations of count fingers, hand motion, or light perception vision. Limited conclusions can be drawn from these levels of VA. Additionally, regarding the management of these cases, many patients were handled very differently during the height of the pandemic with limited operating room access making it difficult to interpret the findings on initial management strategies and final outcomes. Many patients who would have undergone urgent PPV may have instead undergone a tap and inject due to limitations during the early phase of the pandemic, which possibly affected VA.

In summary, our study found that fewer patients presented with endophthalmitis of any etiology during the first six months of the COVID-19 pandemic compared to the same six-month period the year prior. Although clinical characteristics and the post-injection endophthalmitis rate were similar during both periods, patients presented with worse vision during the COVID-19 pandemic suggesting that the pandemic may have contributed to delayed presentation. As the pandemic has drastically impacted the practice of ophthalmology, it is critical that ophthalmologists are prepared to diagnose and treat patients with more severe pathology who may have delayed presentation due to a variety of pandemic-related factors, including fear of exposure, which continues as the pandemic waves ebb and flow.

##  Ethical Considerations

The study protocol was reviewed and approved by the Institutional Review Board at Mayo Clinic, Rochester, MN, USA and under approval number 22-000845.

##  Financial Support and Sponsorship

None.

##  Conflicts of Interest

Dr. Starr has served on Advisory Boards to Genentech, Alimera Sciences, Regenxbio, and Gyroscope Therapeutics.Other authors report no conflicts of interest.

## References

[B1] The Royal College of Ophthalmologists https://www.rcophth.ac.uk/standards-and-guidance/covid-19-resources/national-government-health-organisations-updates/.

[B2] American Academy of Ophthalmology: ONE Network https://www.aao.org/headline/new-recommendations-urgent-nonurgent-patient-care.

[B3] Wong RLM, Ting DSW, Wan KH, Lai KHW, Ko CH, Ruamviboonsuk P, et al (2020). COVID-19: Ocular manifestations and the APAO prevention guidelines for ophthalmic practices. Asia-Pac J Ophthalmol.

[B4] Rodríguez-Ares T, Lamas-Francis D, Treviño M, Navarro D, Cea M, Lopez-Valladares MJ, et al (2021). SARS-CoV-2 in conjunctiva and tears and ocular symptoms of patients with COVID-19. Vision.

[B5] Arora R, Goel R, Kumar S, Chhabra M, Saxena S, Manchanda V, et al (2021). Evaluation of SARS-CoV-2 in tears of patients with moderate to severe COVID-19. Ophthalmology.

[B6] Azzolini C, Donati S, Premi E, Baj A, Siracusa C, Genoni A, et al (2021). SARS-CoV-2 on ocular surfaces in a cohort of patients with COVID-19 from the Lombardy region, Italy. JAMA Ophthalmol.

[B7] Romano MR, Montericcio A, Montalbano C, Raimondi R, Allegrini D, Ricciardelli G, et al (2020). Facing COVID-19 in ophthalmology department. Curr Eye Res.

[B8] Starr MR, Israilevich R, Zhitnitsky M, Cheng QE, Soares RR, Patel LG, et al (2020). Practice patterns and responsiveness to simulated common ocular complaints among US ophthalmology centers during the COVID-19 pandemic. JAMA Ophthalmol.

[B9] Analysis: Ophthalmology lost more patient volume due to COVID-19 than any other specialty https://eyewire.news/articles/analysis-55-percent-fewer-americans-sought-hospital-care-in-march-april-due-to-covid-19/?c4src=article:infinite-scroll.

[B10] Borrelli E, Grosso D, Vella G, Sacconi R, Querques L, Zucchiatti I, et al (2020). Impact of COVID-19 on outpatient visits and intravitreal treatments in a referral retina unit: Let's be ready for a plausible "rebound effect". Graefes Arch Clin Exp Ophthalmol.

[B11] dell'Omo R, Filippelli M, Virgili G, Bandello F, Querques G, Lanzetta P, et al (2022). Effect of COVID-19-related lockdown on ophthalmic practice in Italy: A report from 39 institutional centers. Eur J Ophthalmol.

[B12] Leng T, Gallivan MD, Kras A, Lum F, Roe MT, Li C, et al (2021). Ophthalmology and COVID-19: The impact of the pandemic on patient care and outcomes: An IRIS® Registry Study. Ophthalmology.

[B13] Pellegrini M, Roda M, Lupardi E, Di Geronimo N, Giannaccare G, Schiavi C

[B14] Poyser A, Deol SS, Osman L, Kuht HJ, Sivagnanasithiyar T, Manrique R, et al (2021). Impact of COVID-19 pandemic and lockdown on eye emergencies. Eur J Ophthalmol.

[B15] Savastano A, Ripa M, Savastano MC, Kilian R, Marchini G, Rizzo S (2021). Impact of the COVID-19 pandemic on ophthalmologic outpatient care: Experience from an Italian tertiary medical center. Ann Med.

[B16] Wickham L, Hay G, Hamilton R, Wooding J, Tossounis H, da Cruz L, et al (2020). The impact of COVID policies on acute ophthalmology services-experiences from Moorfields Eye Hospital NHS Foundation Trust. Eye.

[B17] Yen CY, Fang IM, Tang HF, Lee HJ, Yang SH (2022). COVID-19 pandemic decreased the ophthalmic outpatient numbers and altered the diagnosis distribution in a community hospital in Taiwan: An observational study. PLoS One.

[B18] Agarwal R, Sharma N, Patil A, Thakur H, Saxena R, Kumar A (2020). Impact of COVID-19 pandemic, national lockdown, and unlocking on an apex tertiary care ophthalmic institute. Indian J Ophthalmol.

[B19] Romano F, Monteduro D, Airaldi M, Zicarelli F, Parrulli S, Cozzi M, et al (2020). Increased number of submacular hemorrhages as a consequence of coronavirus disease 2019 lockdown. Ophthalmol Retina.

[B20] Li J, Zhao M, She H, Chandra A (2021). The impact of the COVID-19 pandemic lockdown on rhegmatogenous retinal detachment services-experiences from the Tongren Eye Center in Beijing. PLoS One.

[B21] Patel LG, Peck T, Starr MR, Ammar MJ, Khan A, Yonekawa Y, et al (2021). Clinical presentation of rhegmatogenous retinal detachment during the COVID-19 pandemic: A historical cohort study. Ophthalmology.

[B22] Jung JJ, Chang JS, Oellers PR, Ali MH, Do BK, Tseng JJ, et al (2022). Impact of coronavirus disease 2019 restrictions on retinal detachment: A multicenter experience. Ophthalmol Retina.

[B23] Schranz M, Georgopoulos M, Sacu S, Reumueller A, Reiter GS, Mylonas G, et al (2021). Incidence and surgical care of retinal detachment during the first SARS-CoV-2 lockdown period at a tertiary referral center in Austria. PLoS One.

[B24] Das AV, Dave VP (2021). Effect of lockdown and unlock following COVID-19 on the presentation of patients with endophthalmitis at a tertiary eye center over one year. Cureus.

[B25] Gregori NZ, Feuer W, Rosenfeld PJ (2010). Novel method for analyzing Snellen visual acuity measurements. Retina.

[B26] Rush RB, Rush SW (2021). Outcomes in patients resuming intravitreal anti-vascular endothelial growth factor therapy following treatment delay during the coronavirus-19 pandemic. Retina.

[B27] Douglas VP, Douglas KAA, Vavvas DG, Miller JW, Miller JB (2022). Short- and long-term visual outcomes in patients receiving intravitreal injections: The impact of the coronavirus 2019 disease (COVID-19)-related lockdown. J Clin Med.

[B28] Song W, Singh RP, Rachitskaya AV (2021). The effect of delay in care among patients requiring intravitreal injections. Ophthalmol Retina.

[B29] Stone LG, Grinton ME, Talks JS (2021). Delayed follow-up of medical retina patients due to COVID-19: Impact on disease activity and visual acuity. Graefes Arch Clin Exp Ophthalmol.

[B30] Lum F, Li S, Liu L, Li C, Parke DW, Williams GA (2022). The pandemic is not associated with endophthalmitis decrease after anti-vascular endothelial growth factor injections. Ophthalmology.

[B31] LaCroce SJ, Wilson MN, Romanowski JE, Newman JD, Jhanji V, Shanks RMQ, et al (2019). Moraxella nonliquefaciens and M. osloensis are important moraxella species that cause ocular infections Microorganisms.

[B32] Song G, Liang G, Liu W (2020). Fungal co-infections associated with global COVID-19 pandemic: A clinical and diagnostic perspective from China. Mycopathologia.

[B33] Fossataro F, Martines F, Neri P, Allegri P, Pece A

[B34] Agarwal M, Sachdeva M, Pal S, Shah H, Kumar RM, Banker A (2021). Endogenous endophthalmitis: A complication of COVID-19 pandemic: A case series. Ocul Immunol Inflamm.

